# Improved Ultrasound Localization Microscopy Based on Microbubble Uncoupling via Transmit Excitation

**DOI:** 10.1109/TUFFC.2022.3143864

**Published:** 2022-03-02

**Authors:** Jihun Kim, Mathew R. Lowerison, Nathiya V. Chandra Sekaran, Zhengchang Kou, Zhijie Dong, Michael L. Oelze, Daniel A. Llano, Pengfei Song

**Affiliations:** Department of Electrical and Computer Engineering, and the Beckman Institute for Advanced Science and Technology, University of Illinois Urbana–Champaign, Urbana, IL 61801 USA; Department of Electrical and Computer Engineering, and the Beckman Institute for Advanced Science and Technology, University of Illinois Urbana–Champaign, Urbana, IL 61801 USA; Department of Molecular and Integrative Physiology, and the Beckman Institute for Advanced Science and Technology, University of Illinois Urbana–Champaign, Urbana, IL 61801 USA; Department of Electrical and Computer Engineering, and the Beckman Institute for Advanced Science and Technology, University of Illinois Urbana–Champaign, Urbana, IL 61801 USA; Department of Electrical and Computer Engineering, and the Beckman Institute for Advanced Science and Technology, University of Illinois Urbana–Champaign, Urbana, IL 61801 USA; Department of Electrical and Computer Engineering, and the Beckman Institute for Advanced Science and Technology, University of Illinois Urbana–Champaign, Urbana, IL 61801 USA; Department of Molecular and Integrative Physiology, and the Beckman Institute for Advanced Science and Technology, University of Illinois Urbana–Champaign, Urbana, IL 61801 USA; Department of Electrical and Computer Engineering, and the Beckman Institute for Advanced Science and Technology, University of Illinois Urbana–Champaign, Urbana, IL 61801 USA

**Keywords:** Microbubble (MB) separation, MB uncoupling, null excitation, super-resolution imaging, ultrasound localization microscopy (ULM)

## Abstract

Ultrasound localization microscopy (ULM) demonstrates great potential for visualization of tissue microvasculature at depth with high spatial resolution. The success of ULM heavily depends on robust localization of isolated microbubbles (MBs), which can be challenging *in vivo* especially within larger vessels where MBs can overlap and cluster close together. While MB dilution alleviates the issue of MB overlap to a certain extent, it drastically increases the data acquisition time needed for MBs to populate the microvasculature, which is already on the order of several minutes using recommended MB concentrations. Inspired by optical super-resolution imaging based on stimulated emission depletion (STED), here we propose a novel ULM imaging sequence based on MB uncoupling via transmit excitation (MUTE). MUTE “silences” MB signals by creating acoustic nulls to facilitate MB separation, which leads to robust localization of MBs especially under high concentrations. The efficiency of localization accomplished via the proposed technique was first evaluated in simulation studies with conventional ULM as a benchmark. Then, an *in-vivo* study based on the chorioallantoic membrane (CAM) of chicken embryos showed that MUTE could reduce the data acquisition time by half, thanks to the enhanced MB separation and localization. Finally, the performance of MUTE was validated in an *in vivo* mouse brain study. These results demonstrate the high MB localization efficacy of MUTE-ULM, which contributes to a reduced data acquisition time and improved temporal resolution for ULM.

## I. INTRODUCTION

SUPER-RESOLUTION ultrasound localization microscopy (ULM) is an acoustic analog to optical super-resolution microscopy such as photoactivated localization microscopy (PALM) [1] or stochastic optical reconstruction microscopy (STORM) [2]. It was recently introduced for microvasculature imaging beyond the inherent acoustic spatial resolution limit while conserving the imaging depth of conventional ultrasound. The primary idea of ULM is to localize microbubbles (MBs) flowing in the vascular networks to achieve super-resolution, and then track the localized MBs over time to measure blood flow velocity [3]. ULM improves the conventional ultrasound spatial resolution by approximately tenfold and it showed promising results in various tissues including brain [4], kidney [5], liver [6], and tumor [7]. However, practical implementation of ULM is currently limited by its Achilles’ heel—slow temporal resolution, which is largely attributed to the long data acquisition time that is required to capture adequate, spatially separated MB signals for localization and tracking. Although increasing MB concentration alleviates slow MB perfusion and shortens data acquisition time, it causes more MB signals to overlap and become unlocalizable. As such, developing a method that is highly efficient at localizing MBs under high concentration is essential to accelerate ULM.

To this end, several techniques have been demonstrated to improve the super-resolution imaging speed, including super-resolution optical fluctuation imaging (SOFI)-based contrast-enhanced ultrasound (CEUS) imaging [8], and sparsity-based ultrasound super-resolution hemodynamic imaging (SUSHI) [9]. SOFI-based CEUS imaging use the high-order statistics of the fluctuating MBs’ signal. In contrast, SUSHI exploits the sparsity model by assuming the underlying vasculature consisting of point targets on higher pixel resolution. These techniques showed improvement of temporal resolution while providing a spatial resolution that is similar to ULM. However, because of the absence of MB tracking, these methods fall short of providing blood flow velocity measurements, which can be an important biomarker for various applications [4], [10], [11].

Recently, our group introduced an MB separation method that separates high-concentration MBs in the Fourier domain to facilitate localization of MBs under high concentration [10]. Thanks to the enhanced localization efficacy, MB separation leads to a reduced data acquisition time for ULM. However, by creating subsets of data with different MB flow characteristics, MB separation becomes computationally expensive. In addition, the performance of MB separation may be undermined by complex flow dynamics with high MB concentrations. Compressed sensing-based localization techniques also showed the capability of reducing data acquisition time for ULM under MB high concentration, but it has a similar issue of high computational cost as the MB separation method and it also does not provide blood flow velocity measurement [12].

Inspired by the optical super-resolution technique stimulated emission depletion (STED), in this article, we propose to implement a similar principle of contrast signal suppression in ultrasound imaging to promote MB separation and enhance ULM imaging performance. STED uses two transmissions including a Gaussian-shaped beam followed by a doughnut-shaped beam that depletes the fluorescence excited by the Gaussian beam. The partial depletion preserves a sharp focal spot in the donut center, where a small number of fluorophores are allowed to emit signals that are distinct from the surrounding fluorophores that have been depleted [13]. The distinction between the fluorophores inside and outside the donut center creates separation in fluorescence status in time, which manifests as super-resolution when imaging.

While the principle of STED is straightforward, translating it to ultrasound imaging is not because in ultrasound one needs to overcome the challenge of depleting MB signals, which is not as simple as in optics. For example, to deplete MB signal, one needs to apply high mechanical index (MI) acoustic pulses to disrupt MBs, which is not an efficient process and may cause tissue and probe heating. Also, unlike in optical STED where a fluorophore can be instantly and completely switched to the “OFF” or ground status by the stimulation photon, MB signal emission is less binary (e.g., either “ON” or “OFF”) and even disrupted MBs can emit strong acoustic signals [14]. As such, an alternative approach is necessary for ultrasound imaging to implement the principles of STED.

In this article, we introduce a method named MUTE (MB uncoupling via transmit excitation) that uses the principles of acoustic null generation and subtraction imaging to realize STED in ultrasound. MUTE accomplishes MB depletion in two steps: 1) obtain two images by first transmitting a regular plane wave (PW) without acoustic nulls and then another PW with acoustic nulls and 2) calculate the difference between the two images, and then MBs in the nonnull region will be effectively depleted and MBs in the null region will be enhanced and separated from the ones in the non-null region. Although similar ideas of using acoustic nulls to enhance ultrasound imaging resolution have been reported before, they were implemented on receive apodization which does not actively modulate the acoustic response of MBs by transmit excitation [15], [16]. In addition, these methods suffer from a low signal-to-noise ratio (SNR) due to the significantly reduced signal intensity induced by the processes of Heaviside apodization and subtraction [17]. With MUTE, one can create a similar ON/OFF status of MBs as in STED, which provides the opportunity to spatially separate MBs that are otherwise inseparable under traditional insonification. By promoting MB separation, MUTE facilitates robust MB localization for ULM, especially under high MB concentrations. The improved MB localization efficacy leads to an overall reduced data acquisition time for ULM.

The rest of this article is structured as follows: MUTE imaging sequence, postprocessing steps, and preparation of models for evaluation including simulation, chorioallantoic membrane (CAM) of a chicken embryo, and a mouse brain are presented in Section II. In Section III, we will demonstrate the improvement of MUTE-based ULM imaging with conventional ULM as reference. Finally, our results are discussed, and conclusions are drawn in Section IV.

## II. MATERIALS AND METHODS

### A. Principles of Microbubble Uncoupling via Transmit Excitation

**Fig. 1.**
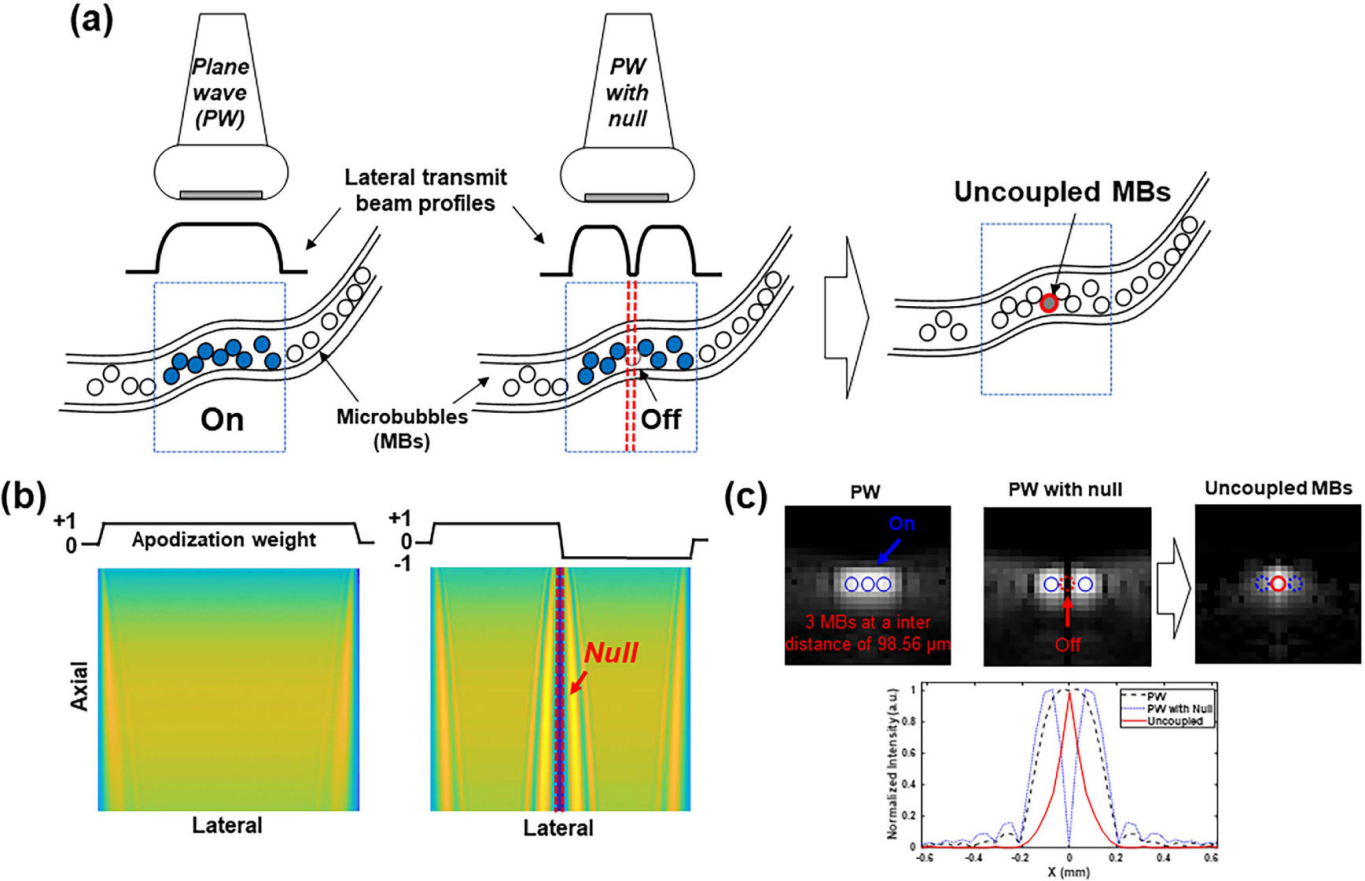
Overall concept of MUTE imaging. (a) Schematic for MBs’ uncoupling. (b) Beam patterns of a PW (left) and PW with null (right) by adjusting the transmit apodization weight. (c) B-mode images of three MBs spaced with 1.28λ (98.56 *μ*m) based on PW (left) and PW with null (middle). λ is the wavelength that was calculated using a transmit frequency of 20 MHz. Uncoupled MBs obtained by calculating the difference between PW image and PW with null image. The rigid and dashed circles in (c) represent the “ON” and “OFF” status of MBs, respectively. The bottom row in (c) shows the normalized intensity profiles in the lateral direction corresponding to each B-mode image in the top row.

Fig. 1(a) shows the conceptual schematic of MB uncoupling in tissue vasculature based on MUTE. Flowing MBs in vessels are activated or silenced depending on their spatial location with respect to the acoustic null. By calculating the difference between two consecutive images (i.e., an image with the acoustic null and the other without), MBs in the non-null region will be suppressed, and MBs in the null region will be enhanced and subsequently uncoupled from the surrounding MBs. In general, images with acoustic nulls can be generated by apodizing the transmit aperture with functions such as Heaviside [15], for example,

(1) $$A_{\text{Tx},i}=\{\begin{array}{l} +1:1\leq i<\frac{N}{2}\\ -1:\frac{N}{2}\leq i<N \end{array}$$

where *A*_Tx*,i*_ is the transmit apodization weight for the *i*th element, and *N* is the total number of active elements in the transmit aperture. Fig. 1(b) shows the beam pattern of PW (left) and MUTE (right) acquired by adjusting the transmit apodization weight. The left and middle panels of Fig. 1(c) show B-mode images of three MBs spaced with 1.28*λ* (98.56 *μ*m) distance, based on PW transmission and MUTE transmission, from which uncoupled MB signals can be obtained by calculating the difference between the two images. *λ* is the wavelength that was calculated using the transmit frequency of 20 MHz. To minimize image distortion by subtraction, an intensity weighted subtraction algorithm was adopted for the MUTE sequence [18]

(2) $$I_{\text{uncoupled}\;}=I_{\text{PW}}-\alpha \cdot I_{\text{MUTE}}.$$

(3) $$\alpha =\frac{\left(I_{\text{PW}}-I_{\text{Null}}\right)-\varepsilon_{\text{min}}}{\varepsilon_{\max}+\varepsilon_{\min}}.$$

where *I*_PW_ and *I*_MUTE_ are the intensity images (i.e., B-mode) obtained by PW and MUTE, respectively. *ε*_max_ and *ε*_min_ are the upper and lower bounds of the intensity values of the uncoupled MB image, respectively, in which we set −1 and +1, as the minimum and maximum intensity values. The bottom row in Fig. 1(c) shows the normalized intensity profiles in the lateral direction corresponding to each B-mode image. One can observe that the center MB in the acoustic null that was not distinguishable from the surrounding MBs now becomes distinct and localizable for ULM imaging.

### B. MUTE Imaging Sequence and Data Acquisition

**Fig. 2.**
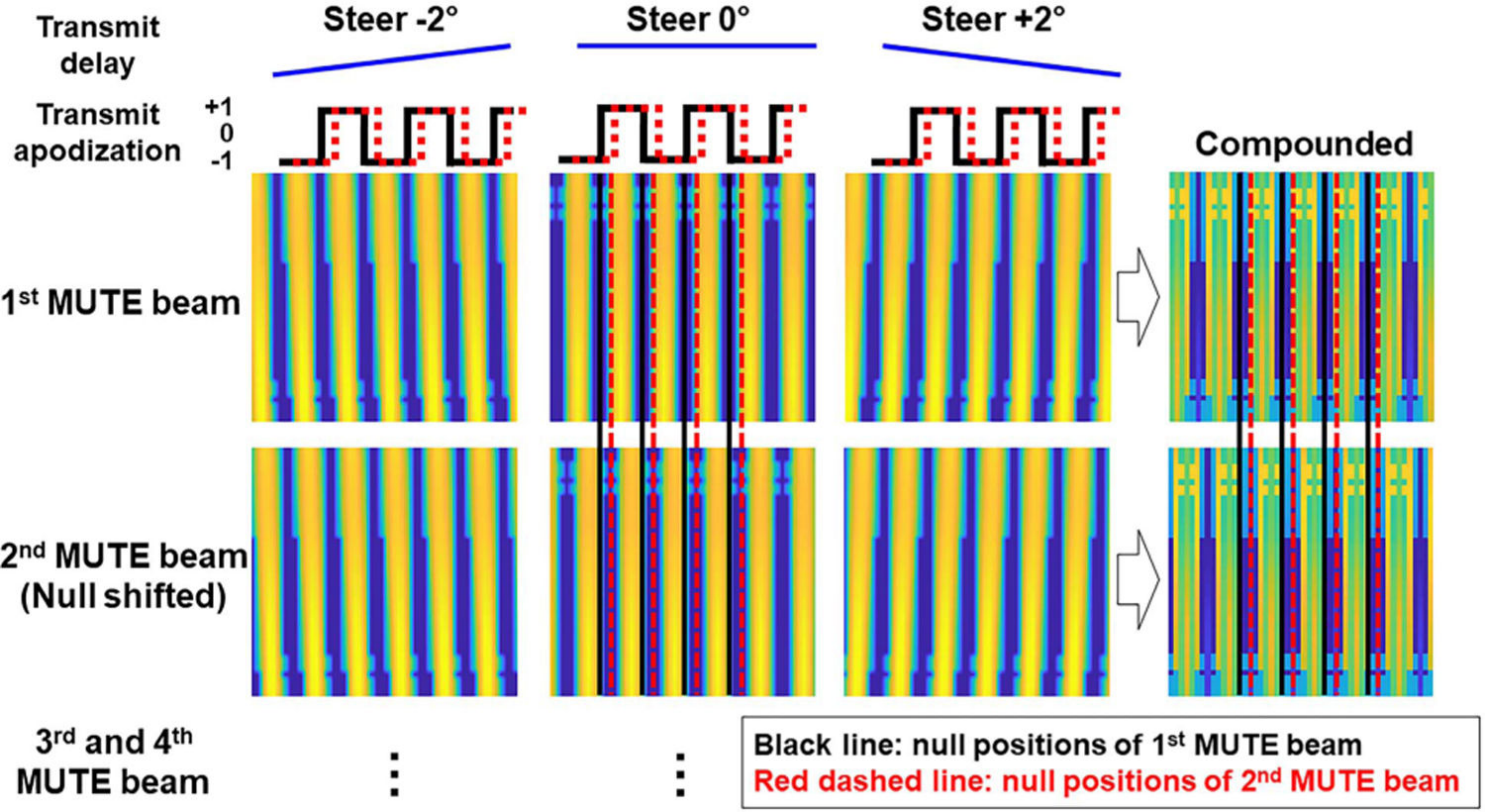
MUTE imaging sequence. Beam patterns of four MUTE sequences with three steering angles of −2°_, 0_°_, and_ +2°_. Each MUTE_ sequence has different acoustic null positions as indicated by the black and red dashed lines depending on transmit apodization shifting.

In practice, because a single acoustic null only uncouples MBs in a small-limited region, it becomes challenging to implement MUTE with an adequate frame rate for ULM imaging (e.g., one needs to translate the null to different lateral locations in different pulse-echo cycles). Also importantly, since a single PW does not provide transmit focusing and adequate imaging SNR, it is necessary to integrate spatial angular compounding into the MUTE sequence for practical use [19]. To address these practical issues, as shown in Fig. 2, we designed a pulse train of Heaviside functions for transmit apodization, which was then steered with various angles to realize spatial compounding by adjusting the transmit delay for each element. In detail, the MUTE beam includes a total number of 31 nulls with equal distances of 3.58*λ* (275.7 *μ*m) in the lateral direction. The width of null is 0.45*λ* (34.6 *μ*m). Furthermore, to cover the entire imaging field by nulls, the MUTE beam was translated laterally with a step size of 0.89*λ* (68.5 *μ*m) corresponding to the pitch size of the transducer we used, resulting in the use of four MUTE beams at each steering angle. To minimize overlap of non-null regions between different steering angles while still providing ample angular compounding, a step steering angle of 2° was chosen throughout the study. As shown in Fig. 2, upon spatial compounding of each MUTE sequence, the acoustic nulls were preserved while the non-null regions were compounded with enhanced transmit focusing and SNR.

For the acquisition of ultrasound in-phase and quadrature (IQ) data, we used an ultrasound imaging system (Vantage 256, Verasonics Inc., Kirkland, WA, USA) with a high-frequency linear array transducer (L35–16vx, Verasonics Inc., Kirkland, WA, USA). The receiving demodulation frequency was set as 31.25 MHz. The transmit frequency of 20 MHz with a single sinusoidal wave and a transmit voltage of 10 V were used. Furthermore, ultrasound imaging was performed using three steering angles with a step steering degree of 2 (−2°, 0°, +2°). PW and four MUTE beams were sequentially generated at each steering angle, and coherent compounding was performed separately to each IQ sub-datasets. The postcompounded effective frame rate was 500 Hz. The total number of 5000 IQ datasets including each 1000 IQ subdatasets for PW and four MUTE sequences were stored per one acquisition, which corresponds to the data acquisition time of 2 s.

To measure the beam pattern and MI generated by the MUTE sequence, a needle hydrophone (1603, Precision Acoustic Ltd., Dorset, U.K.) with a customized acoustic intensity measurement system was used.

### C. Postprocessing Steps for the Reconstruction of MUTE-ULM Image

**Fig. 3.**
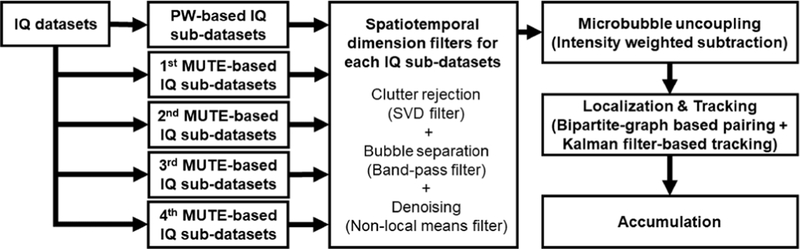
Postprocessing steps for reconstruction of MUTE-ULM image including clutter rejection, MB separation, denoising, MB uncoupling, localization, and tracking. These processes were repeated for each imaging frame, and then the final ULM image was reconstructed by accumulation of MB tracks.

For the reconstruction of MUTE-ULM image, the postprocessing steps are as shown in Fig. 3. A spatiotemporal singular value decomposition (SVD) filter was used to extract the MB signal from the surrounding tissue [20], [21]. A noise equalization process was applied throughout the imaging field of view to facilitate robust thresholding based on MB signal intensity [22]. MB separation was used in this study with three filter bands of 15– 50, 50–100, and 100–200 Hz [23]. After MB separation, a spatiotemporal nonlocal means (NLM) filter was used to further reduce noise that may result in false MB localizations [24]. We then performed MUTE image subtraction based on (2) followed by MB localization and tracking. For MB localization, MB images were spatially interpolated to 0.06*λ* (4.9 *μ*m) pixel size. Two point-spread functions (PSFs) were then constructed for PW and MUTE sequences, respectively, using a 2-D multivariate Gaussian model

(4) $${\text{PSF}}_{\text{MBs}}=\exp\left(-\left(\frac{{\left(z-c_{z}\right)}^{2}}{2\sigma_{z}^{2}}+\frac{{\left(x-c_{x}\right)}^{2}}{2\sigma_{x}^{2}}\right)\right)$$

where (*c*_*z*_*, c*_*x*_) represents the center location of MB. *σ*_*z*_ and *σ*_*x*_ were calculated in the axial and lateral directions, respectively, as follows:

(5) $$\sigma =\frac{\text{FWHM}}{2\sqrt{2\ln2}}.$$

where the full-width at half-maximum (FWHM) of MBs was measured by averaging 10 random MB signals obtained at different imaging depths and frames. In this study, the average FWHM of MBs via PW in the lateral and axial directions was measured to be 138 and 86 *μ*m, respectively; and 120 and 86 *μ*m for MUTE. Note, that the null width of MUTE beam was measured to be ~50 *μ*m.

After constructing appropriate PSFs, a similar process of MB localization and tracking as in our recent papers [24], [25] was performed.

### D. Simulation Study

To evaluate the efficiency and error of localization depending on the concentration of MBs, we produced digital phantoms with randomly distributed MBs at concentrations of 5, 25, 50, 100, 125, and 150 MBs/mm^2^ using the Verasonics simulator. Furthermore, five phantoms were generated at each concentration to measure the average performance with standard deviations. The same MUTE imaging sequence as described in Fig. 2 was used in the simulation study. Besides, the same postprocessing steps were applied except for spatiotemporal dimension filters. The localized MBs were paired within 1*λ* (77 *μ*m) distance with the true MBs generated in simulation using a bipartite graph-based pairing algorithm [24]. Then, the localization efficiency was measured by counting the number of pairings at each concentration. Simultaneously, the localization error was calculated by measuring the distance between paired MBs.

### E. Chicken Embryo Chorioallantoic Membrane (CAM) Study

In this section, we used CAM microvessel with optical imaging ground truth to test the imaging performance of MUTE. Fertilized chicken eggs (white leghorn) were obtained from the University of Illinois Poultry Research Farm and stored in a 37 °C egg incubator (Digital Sportsman Cabinet Incubator 1502, GQF Manufacturing Inc., GA, USA). On the fourth day of embryonic development (EDD-04), the eggshells were peeled using a rotary Dremel tool with a cutting wheel, and embryos were transferred into weigh boats. Shell-less embryos were stored in a humidified incubator (HH09, Darwin Chambers Company, MO, USA) until the 16th day of embryonic development (EDD-16) to perform ultrasound imaging.

Food and Drug Administration (FDA)-approved lipid shell MBs (Definity^®^, Lantheus Medical Imaging Inc., N. Billerica, MA, USA) were used throughout *in vivo* imaging experiment. The final solution of MBs had a concentration of 1.2 × 10^10^ MBs/mL [26]. For MB injection, a high-order vein on the CAM was cannulated using a glass capillary needle to allow MBs’ bolus injection of 70 *μ*L (8.4 × 10^8^ MBs) intravenously. Then, the ultrasound transducer was placed at the side of the weigh boat. An optical image of the CAM vessels was acquired using a stereomicroscope (SMZ800N, Nikon Co., Tokyo, Japan) to provide the reference ground truth for imaging performance evaluations. For reconstruction of the ULM image of CAM, a total of 20 IQ datasets corresponding to 20 000 frames were acquired based on the ultrasound imaging setup we described in previous sections.

To measure the ULM imaging performance between PW and MUTE sequences, a normalized cross correlation (NCC) was computed between ULM image and optical image and used as a quantitative measure of similarity. To accomplish this, the optical image was first resized according to the dimension of the ULM image and then converted to grayscale. Then, a target patch with a size of 98 *μ*m × 98 *μ*m (20 pixels × 20 pixels) was selected from the optical image as the reference. A smaller patch (24.5 *μ*m × 24.5 *μ*m or 5 pixels × 5 pixels) was then selected from ULM image for NCC calculation This process was repeated for the entire imaging FOV (patch translation step size = 4.9 *μ*m or 1 pixel), and the average NCC coefficients was used as the quantitative measurement of ULM imaging performance. In addition to similarity, we measured the vessel filling (VF) percentage for each imaging sequence. VF is defined as:

(6) $$\text{VF}(\%)=\frac{N_{\text{ULM}}}{N_{\text{GT}}}\times 100.$$

where *N*_GT_ denotes the total number of true vessel pixels, and *N*_ULM_ is the total number of pixels that have been correctly filled by ULM using MB locations. To obtain *N*_GT_, Otsu’s thresholding was applied to grayscale optical image followed by image dilation and erosion to extract vascular morphometric information.

### F. In Vivo Mouse Brain Study

All experimental procedures on the mouse presented in this article were approved by the IACUC at the University of Illinois Urbana-Champaign (Protocol number: 19063). Anesthesia was induced using 4% isoflurane mixed with medical oxygen in a gas induction chamber. The mouse was then transferred to a nose cone supplying 2% isoflurane with oxygen for maintenance, and its head was secured to a stereotaxic imaging frame with ear bars. The scalp was removed with surgical scissors, and a cranial window on the left side of the skull was opened with a rotary Dremel tool to expose a hemisphere of the brain. The ultrasound transducer was fixed to a customized stereotaxic imaging stage and positioned above the cranial window to image the brain with a coronal imaging plane. A 30-gauge catheter was then inserted into the tail vein of the mouse for 50-*μ*L MB injection (6 × 10^8^ MBs). Additional 50-*μ*L booster injections were provided throughout the imaging session to maintain MB concentration in the cerebral vasculature. For reconstruction of the ULM image of the mouse brain, a total of 40 IQ datasets corresponding to 40 000 frames were obtained based on the ultrasound imaging setup we described in previous sections during two bolus injections.

## III. RESULTS

### A. Measurement of the Beam Pattern and Mechanical Index of MUTE Sequence

**Fig. 4.**
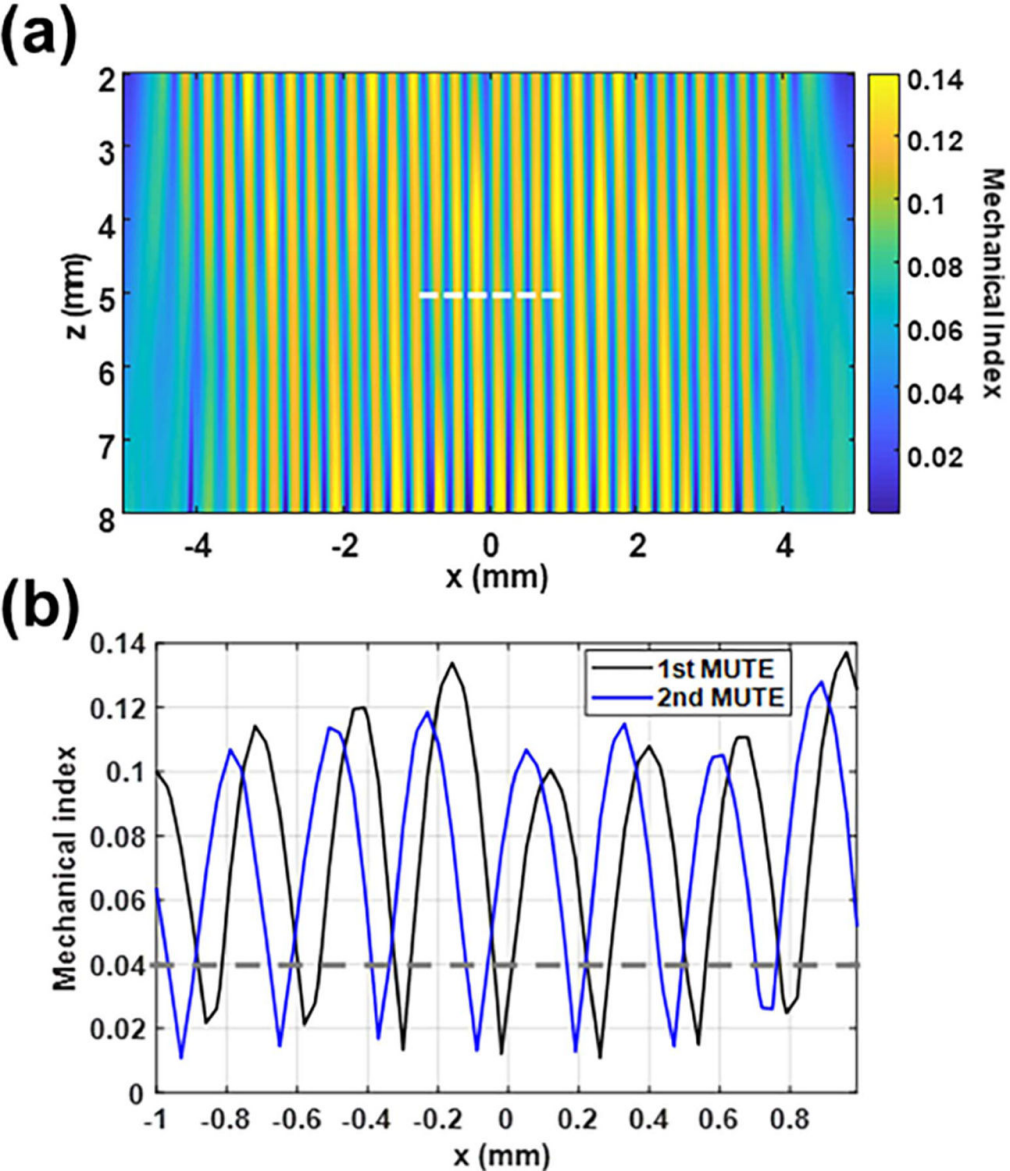
Measurement of the beam pattern and MI of MUTE beam with a transmit frequency of 20 MHz and a transmit voltage of 10 V at a steering angle of 0° using a needle hydrophone. (a) Representative MUTE beam pattern and (b) MI profiles of first and second MUTE beams acquired at a depth of 5 mm, as indicated by the white dashed line in (a). The gray dashed line in (b) indicates the baseline for measuring the width of null. Nulls were detected every 3.64*λ* (280 *μ*m) with a width of 0.65*λ* (50 *μ*m) in the lateral direction. The nulls were shifted laterally with a step size of 0.91*λ* (70 *μ*m).

We measured the acoustic null width, shift, and distance between the nulls of a MUTE beam using a needle hydrophone. The MUTE beam was generated with a transmit frequency of 20 MHz and a transmit voltage of 10 V and at a steering angle of 0°. Fig. 4(a) shows the 2-D MI image of the 1st MUTE beam. The needle hydrophone measurement confirmed the specifications of the designed MUTE beam: 31 null positions with an inter distance of 3.64*λ* (280 *μ*m) in the lateral direction. The maximum MI was measured to be 0.14. Fig. 4(b) represents the lateral MI profiles acquired from the first (Black) and second (blue) MUTE beams at 5 mm in depth indicated by the white dashed line in Fig. 4(a). The width of null was measured at the MI of 0.04 indicated by a gray dashed line in Fig. 4(b). The average width of nulls was measured to be around 0.65*λ* (50 *μ*m). Furthermore, it was confirmed that null locations of the MUTE beam could be laterally moved 0.91*λ* (70 *μ*m) [Fig. 4(b)].

### B. Evaluation of MUTE-Based Localization Efficacy in Simulation

**Fig. 5.**
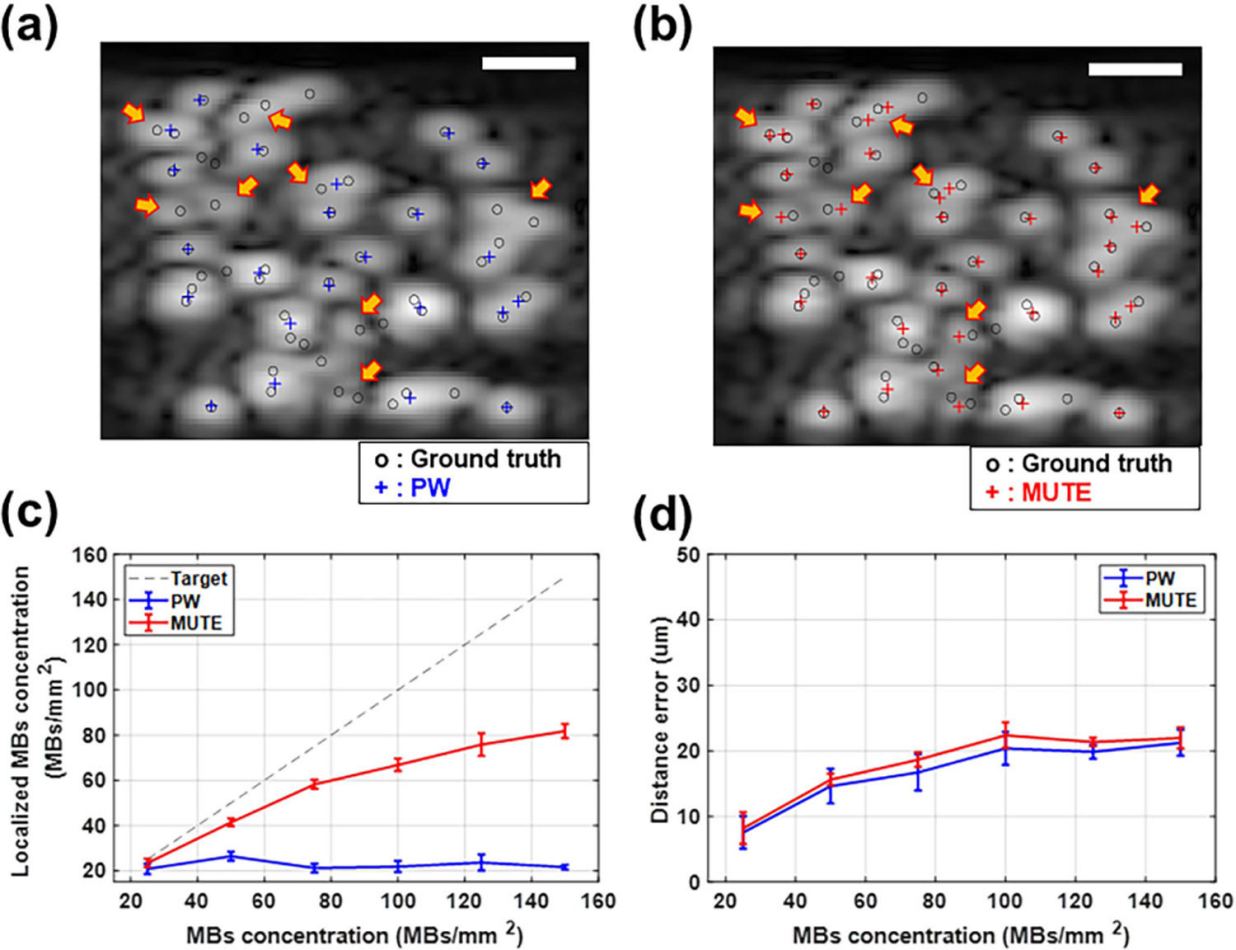
Evaluation of localization efficacy and error using the randomly distributed MBs with different concentrations in simulation: Localization results at a concentration of 50 MBs/mm^2^ overlaid on the B-mode images based on (a) PW and (b) MUTE. Blue and red crosses indicate the localization results of MBs based on PW and MUTE, whereas black circles indicate the true positions of MBs. (c) and (d) Localization efficacy and localization error with different concentrations of MBs, respectively. The gray dashed line in (c) indicates targeted localization efficacy. Scale bars in (a) and (b) represent 200 *μ*m.

We evaluated the performance of PW and MUTE-based localization using different concentrations of simulated MB signals. Fig. 5(a) and (b) shows the representative localization results based on each dataset obtained by PW and MUTE imaging sequences at a concentration of 50 MB/mm^2^. The MUTE-based localization had a higher MB detection rate than PW-based localization, especially for the overlapped MBs indicated by yellow arrows in Fig. 5(a) and (b). The average number of MB localizations was 26.4 ± 1.96 MB/mm^2^ for PW and 41.4 ± 1.74 MB/mm^2^ for MUTE at a concentration of 50 MB/mm^2^ [Fig. 5(c)]. The MUTE-based localization had higher efficacy with a similar localization error across all the MB concentrations tested [Fig. 5(c) and (d)]. In particular, as shown in Fig. 5(c), MUTE could localize over 80% of MBs up to 50 MB/mm^2^ concentration, whereas PW fell short of reaching 80% beyond 25 MB/mm^2^. Moreover, MUTE was largely capable of keeping up with the elevated MB concentration, while PW plateaued beyond 50 MB/mm^2^ concentration [Fig. 5(c)]. At the maximum concentration of 150 MB/mm^2^, MUTE was still able to localize over 50% of the MBs (81.8 MB/mm^2^*)*. The maximum localization error for both PW- and MUTE-based localization was below 24.64 *μ*m, which corresponds approximately to 0.32*λ* of the imaging frequency [Fig. 5(d)].

### C. MUTE-ULM Imaging of CAM and Quantitative Analysis With Optical Microscopy

**Fig. 6.**
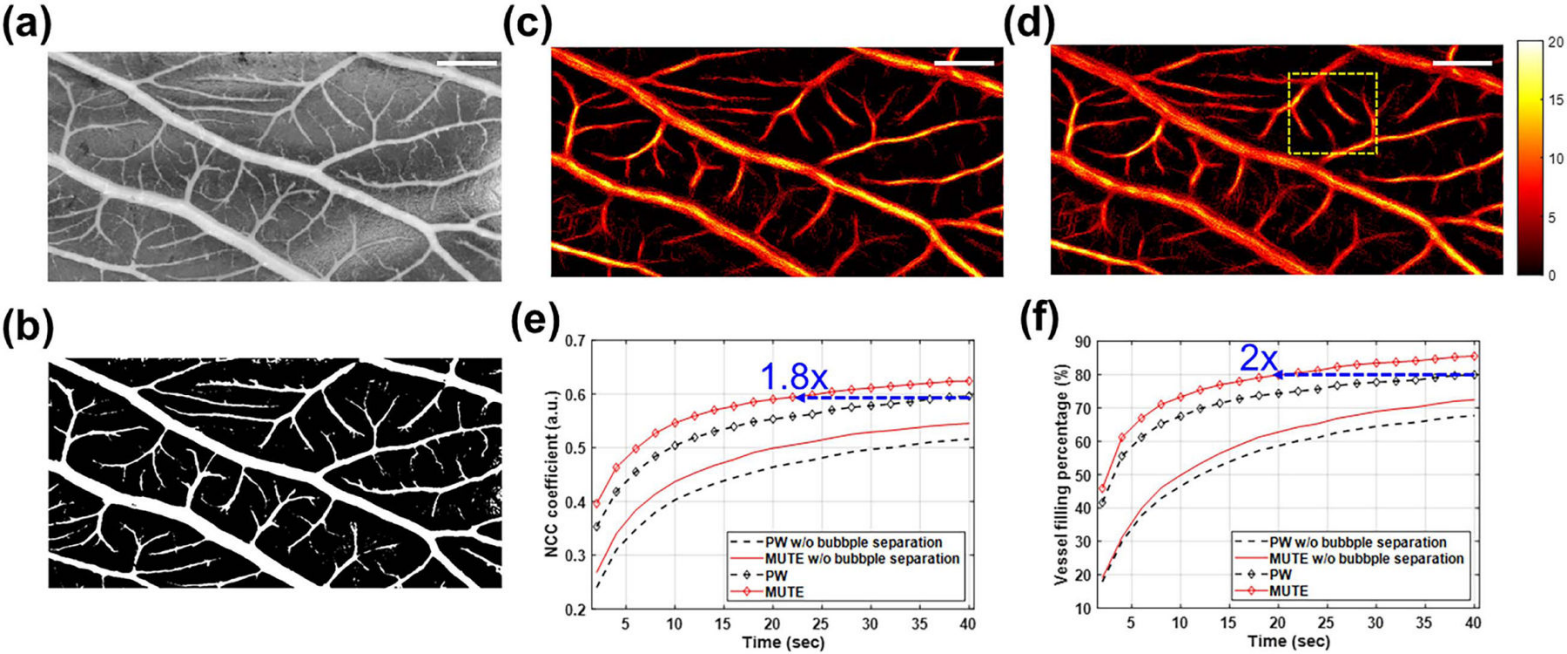
ULM imaging of CAM vessels of chicken embryo validated with optical microscopy. (a) Grayscale optical image, and corresponding (b) binary optical image, (c) PW-, and (d) MUTE-ULM images of CAM vessels. ULM images were represented by the same color scales. (e) Similarity and (f) VF percentage of each ULM image according to data acquisition time, validated using optical binary image of CAM vessels. The red and black dashed lines with markers in (e) and (f) represent the results with filtering-based bubble separation technique. Scale bars represent 500 *μ*m.

The optical image of the CAM in grayscale is shown in Fig. 6(a), and the corresponding binary image generated by Otsu’s thresholding is shown in Fig. 6(b). PW- and MUTE-ULM images are shown in Fig. 6(c) and (d), respectively. These images were reconstructed using 20 000 frames of ultrasound data corresponding to a total acquisition time of 40 s. Fig. 6(e) shows the NCC coefficient between ULM and the optical ground truth versus data acquisition time. It can be observed from Fig. 6(e) that MUTE-ULM generated images with greater similarity to the optical ground truth than did PW-ULM. The maximum NCC coefficient of PW-ULM was 0.59 at an acquisition time of 40 s, while MUTE-ULM achieved the same level of similarity with approximately half the time (22 s). Fig. 6(f) shows the VF percentage of the two techniques, and MUTE-ULM again outperformed PW-ULM across all data acquisition times. MUTE-ULM filled the vessels about twice as fast as did PW-ULM (i.e., 20 s versus 40 s) and was able to reach a higher VF percentage than PW-ULM (85.5% versus 79.9%). Furthermore, it was observed that the maximum NCC coefficient (i.e., a measure of similarities between the optical and ULM images of CAM) was improved from 0.51 to 0.55 from PW to MUTE without bubble separation, and from 0.51 to 0.59 from PW to PW with bubble separation. The VF percentage was enhanced from 67.6% to 72.4% from PW to MUTE without bubble separation, and from 67.6% to 79.9% from PW to PW with bubble separation [Fig. 6(e) and (f)].

**Fig. 7.**
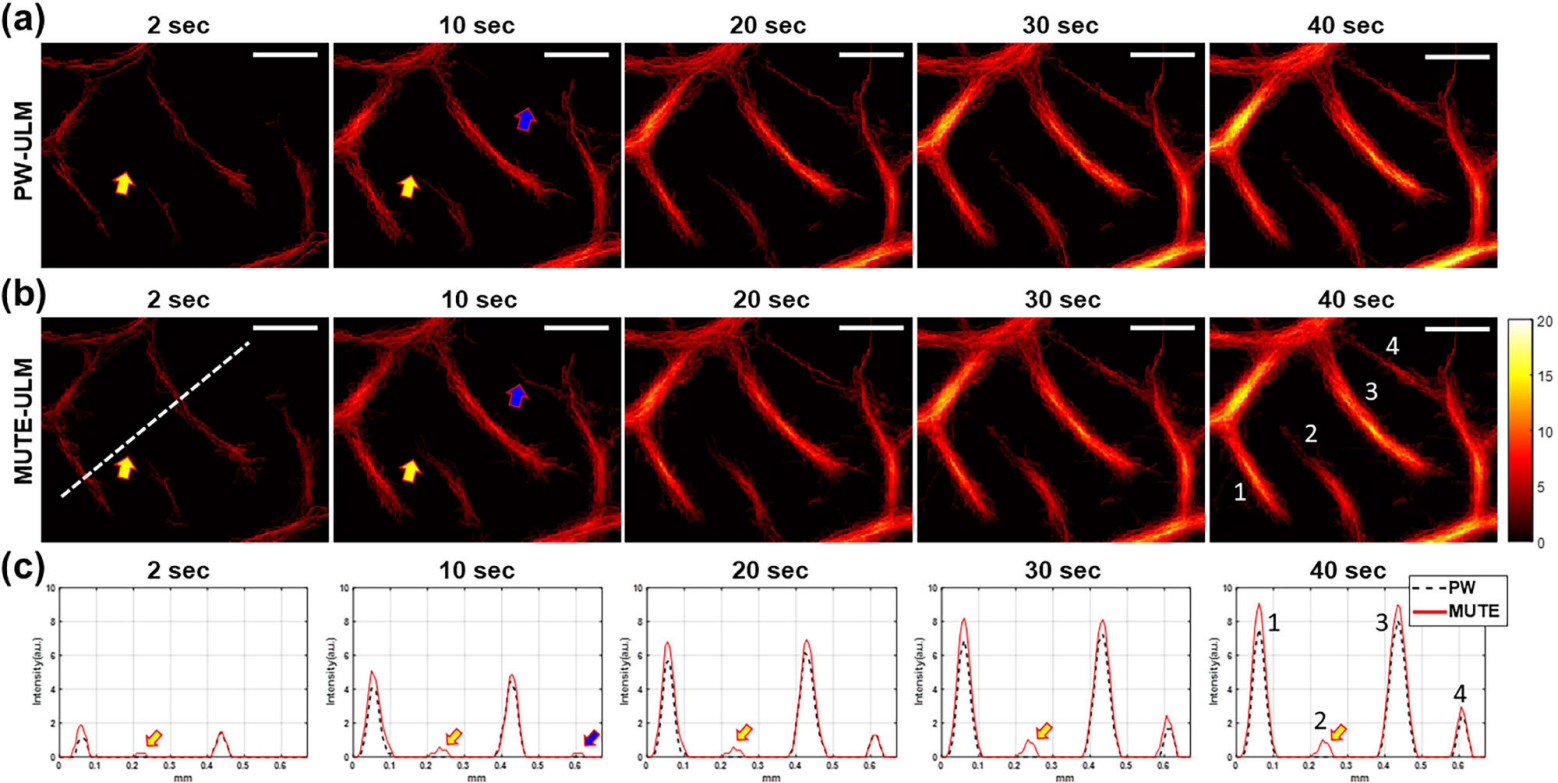
Local analysis of (a) PW- and (b) MUTE-ULM images. The images were extracted from regions indicated by the yellow dashed box in Fig. 6(d). (c) 1-D plots of vessel signal at different accumulation times from the location indicated by white dashed line in (b). Different colored arrows indicate different examples of vessels that demonstrate faster VF speed of MUTE-ULM. Scale bars represent 200 *μ*m.

Fig. 7 shows the local analysis results of ULM images in the yellow box in Fig. 6. As indicated by the different colored arrows in Fig. 7(a) and (b), MUTE-ULM demonstrated faster VF speed and higher overall MB count [Fig. 7(c)] than PW-ULM. MUTE-ULM could detect small vessel signals starting from 2 s, while PW-ULM could not detect the small vessels properly at the same acquisition time. As indicated by yellow arrows in Fig. 7(c), the second vessel was only detectable with MUTE-ULM and remained missing with PW-ULM until 40 s. Moreover, the fourth vessel indicated by the blue arrow was detected by MUTE-ULM at *t* = 10 s [Fig. 7(c)], detected the same vessel at *t* = 20 s. Furthermore, we compared the diameter of the four vessels that were measured at 40 s as labeled in Fig. 7(c). Based on MUTE-ULM, the diameter of vessels was measured to be 40.6, 29.7, 52, and 32 *μ*m, respectively, for vessels 1–4, while the diameter of vessels was 39.23, 0 (missing), 50, and 30.77 from PW-ULM. The diameters measured from the optical ground-truth image were 36.8, 25.6, 42.7, and 28.8 *μ*m. The average error in diameter measurement was 5.1 *μ*m for MUTE-ULM and 9.33 *μ*m for PW-ULM.

### D. MUTE-ULM Imaging of the Mouse Brain in Vivo

**Fig. 8.**
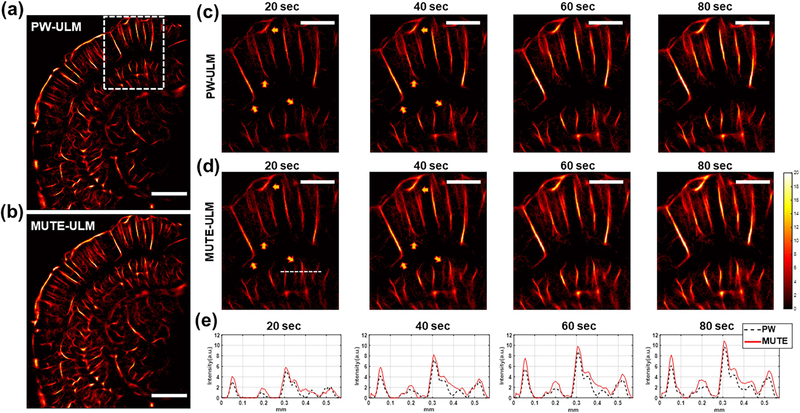
ULM imaging of a mouse brain *in vivo*. (a) PW- and (b) MUTE-ULM images of mouse brain. Scale bars in (a) and (b) represent 1 mm. Local analysis of (c) PW- and (d) MUTE-ULM images. The images were extracted from regions indicated by the white box in (a). (e) 1-D plots of vessel signal at different accumulation times from the location indicated by white dashed line in (d). The yellow arrows indicate different examples of vessels that demonstrate faster filling speed of MUTE-ULM. Scale bars in (c) and (d) represent 500 *μ*m. Every image was generated in the same color scale.

Fig. 8 shows the ULM imaging results using MUTE and conventional PW on a mouse brain. A total of 40 000 frames (80 s) of ultrasound data were acquired during the experiment. We also carried out the local analysis at the regions indicated by the white box in Fig. 8(a). From Fig. 8, both PW- and MUTE-ULM were able to produce robust, super-resolved brain microvasculature maps throughout the full depth of the brain. MUTE-ULM showed higher intensity microvessel density maps that indicate higher MB count, which is most conspicuous in regions with smaller vessels between large vessels. Similar to the observations from CAM study, MUTE-ULM showed a faster VF speed than PW-ULM, as indicated by the yellow arrows in Fig. 8(c) and (d). The intensity profiles of vessels through the white dashed line indicated in Fig. 8(d) demonstrate that MUTE-ULM detected a higher MB count than PW-ULM, which is again consistent with the results in the CAM study. These results, thus, demonstrate that the proposed technique has the potential to shorten the data acquisition time for *in vivo* applications.

**Fig. 9.**
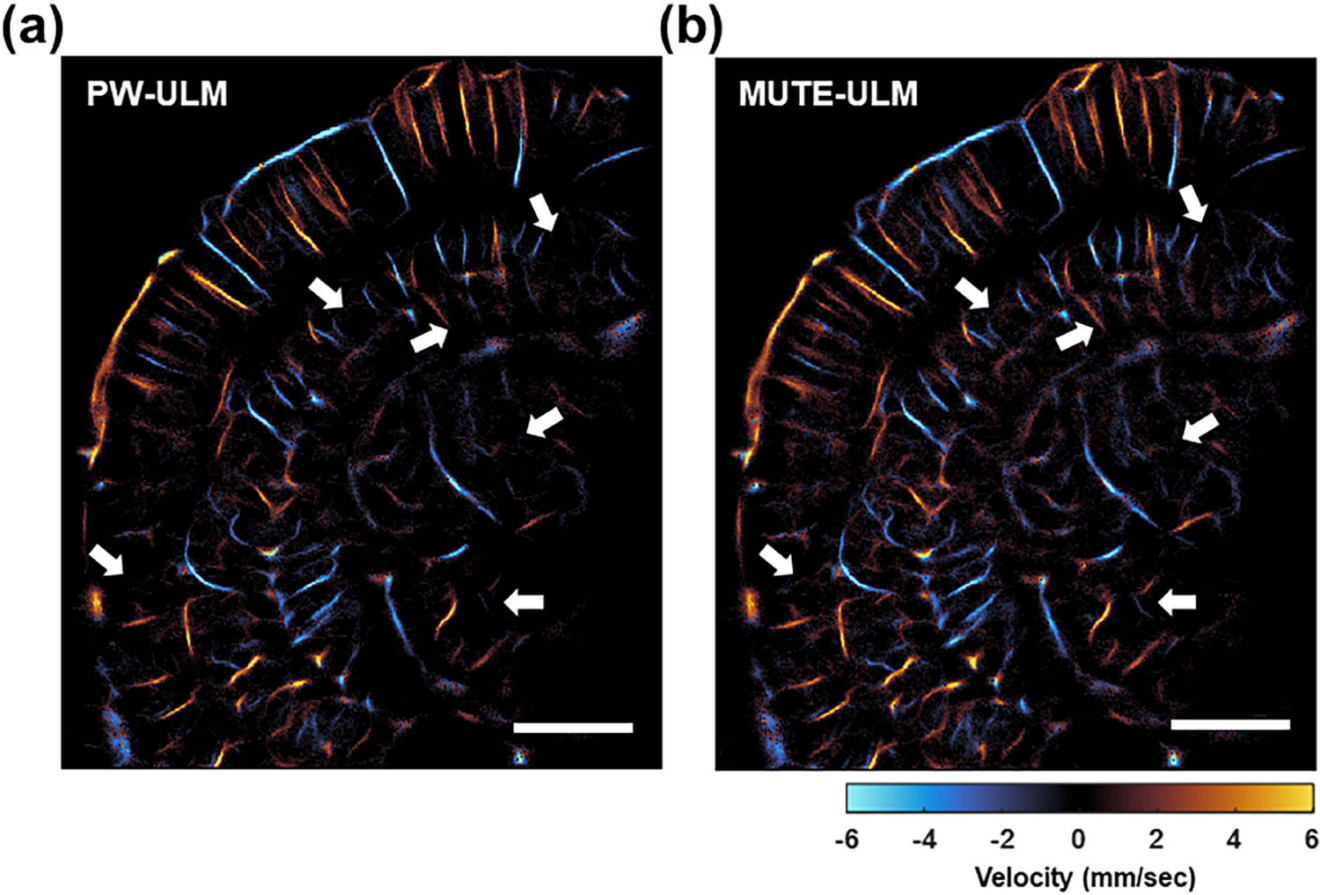
Velocity map of mouse brain reconstructed based on (a) PW- and (b) MUTE-ULM. MUTE-ULM offers clearly better performance as indicated by white arrows in (a) and (b). Scale bars in (a) and (b) represent 500 *μ*m.

The velocity maps of mouse brain reconstructed via PW- and MUTE-ULM using entire datasets (80 s) are shown in Fig. 9(a) and (b). MUTE-ULM does not affect the measurement of the velocity map while assuring better detectability for the localization of high-concentration MBs as denoted by white arrows in Fig. 9(a) and (b).

## IV. DISCUSSION

In this article, we introduced an acoustic analog to STED for super-resolution ultrasound microvascular imaging. MUTE enhances the performance of ULM by uncoupling overlapping MB signals in space based on acoustic nulls and image subtraction. We demonstrated that MUTE significantly improved the performance of ULM, especially under high MB concentrations. This has strong implications for the practical implementation of ULM because a higher MB concentration leads to a faster vascular filling time, which then translates to shorter data acquisition and higher temporal resolution for ULM.

Unlike in STED where optical fluorophores can be conveniently turned on or off by different wavelength emissions, MBs have a less binary response to acoustic waves and are difficult to transition between the ON and OFF status. Although high MI acoustic pulses can disrupt MBs and switch them OFF, the disruption process takes a long time and even the disrupted MBs (i.e., free gas) can be echogenic. Therefore, in this article, we resorted to using acoustic nulls and the principles of image subtraction to realize STED for ultrasound super-resolution imaging. Although the concept of using acoustic nulls to enhance ultrasound imaging resolution was reported before (e.g., NSI), these methods exclusively operated in the receive beamforming process and did not actively modulate MB response with the acoustic nulls.

In the simulation study, the nonuniform distribution at both sides of the null was observed when the full transducer aperture was used for null creation (i.e., transmit apodization: +1 for the left 64 elements and −1 for the right 64 elements), which induced high-level sidelobes [e.g., blue dashed line of the intensity profile in Fig. 1(c)]. However, as shown in Fig. 1(c), the side lobes could be effectively suppressed (e.g., red line of the intensity profile) using the intensity weighted subtraction algorithm [(2) and (4)]. Furthermore, the side lobes became less pronounced when using the sub-aperture technique (e.g., as shown in Fig. 4 where subapertures of eight elements were used for null generation), which was confirmed by hydrophone measurements.

We first evaluated the performance of MUTE *in silico* with randomly distributed MBs. We found that MUTE-based localization was highly effective with uncoupling the spatially overlapped MBs that are otherwise inseparable and unlocalizable by conventional ULM. MUTE also contributes to a more accurate MB localization when compared with conventional ULM [Fig. 5]. We then tested the performance of MUTE *in vivo* on a chicken embryo CAM model [Figs. 6 and 7] and a mouse brain [Figs. 8 and 9] with high concentration MB injections. In both the models, MUTE-ULM demonstrated significantly faster VF time and higher MB count than conventional ULM. These results suggest that MUTE can effectively mitigate the issue of slow imaging speed of ULM by promoting a more robust MB localization with high efficacy under high MB concentrations, which translates to a faster vascular MB filling rate and an overall reduced data acquisition time.

**Fig. 10.**
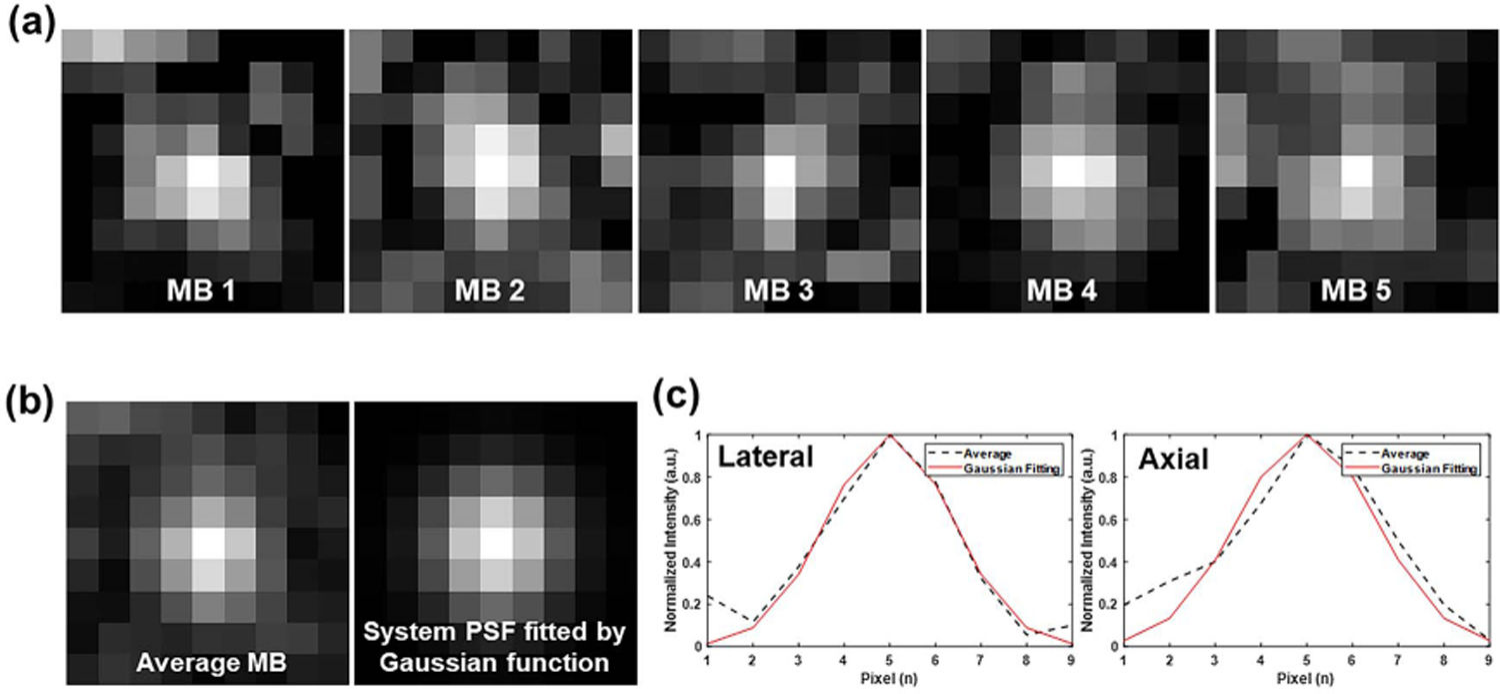
Examination of PSF by MUTE-ULM from CAM and mouse datasets. (a) Examples of uncoupled MB images acquired randomly. (b) Averaged MB image based on ten uncoupled MB images (left) and the system PSF fit by Gaussian function (right). (c) Lateral (left) and axial (right) profiles of (b). The black dashed lines were acquired from the averaged MB, while the red lines were acquired from the constructed PSF.

The lower precision of localization via the wrong system PSF could lead to an over-estimation of the diameter of the vessel (or blurring) in ULM image. In this study, we randomly selected 10 uncoupled MB images to derive a PSF to be used for MB localization as described in Section II–C. For example, five representative MBs after uncoupling by MUTE are shown in Fig. 10(a). Fig. 10(b) shows the averaged MB signal, which does resemble a 2-D Gaussian function [further confirmed by the 1-D axial and lateral profile plots in Fig. 10(c)]. One possible reason for the slightly increased localization error for MUTE is the distortion of the MB signal (i.e., from null subtraction) that results in a slight shift of the detected MB location from the true location. Nevertheless, the error was only slightly higher [e.g., 0.017*λ* (1.3 *μ*m) in Fig. 5(d)] than conventional ULM and the maximum error was under 0.32*λ* (24.64 *μ*m), which still assures the capability of super-resolution imaging over the diffraction limit.

**Fig. 11.**
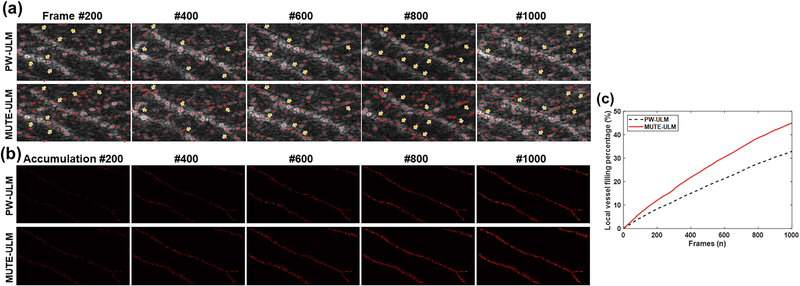
Comparison of localization performance of PW- and MUTE-ULM at the large vessel of CAM validated with optical microscopy. (a) Representative localization results overlaid on the B-mode image of CAM at each frame. The red cross represents localized MBs. MUTE-ULM shows the higher efficiency for the overlapped MBs as denoted by yellow arrows. (b) Accumulation map of localized MBs without tracking depending on the number of frames we used. The vessels were manually segmented under the examination of aligned optical image of CAM. (c) VF percentage at large vessels according to the accumulated number of frames.

The computational cost of MUTE-ULM including beamforming and post-processing is indeed fivefold more than PW-ULM. Beyond the postprocessing cost limitation, the low frame rate caused by the null transmission sequences is a limitation of MUTE-ULM in comparison to PW-ULM. One can acquire more PW data with a higher frame rate to accumulate more MB signals to improve PW-ULM performance. Besides, a higher frame rate is also helpful for better tracking results of MBs. However, the capability of localizing MBs in vessels with high MB concentrations does not necessarily improve with increased imaging frame rate or increased MB data. This is particularly true in larger vessels with high MB concentrations. As shown in Fig. 11(a), MUTE had much better performance in MB localization in the two parallel major vessels than PW-based ULM. Moreover, we plotted representative localization results using 200, 400, 600, 800, and 1000 frames for both MUTE-ULM and PW-ULM [Fig. 11(b)]. We examined the VF percentage at large vessels, which clearly shows that MUTE-ULM had a higher VF rate than conventional PW-ULM [Fig. 11(c)]. Combining these results with the observation of higher MB localization efficacy, the gain in ULM imaging performance from MUTE is primarily a result of enhanced MB localization instead of an acquired larger dataset.

**Fig. 12.**
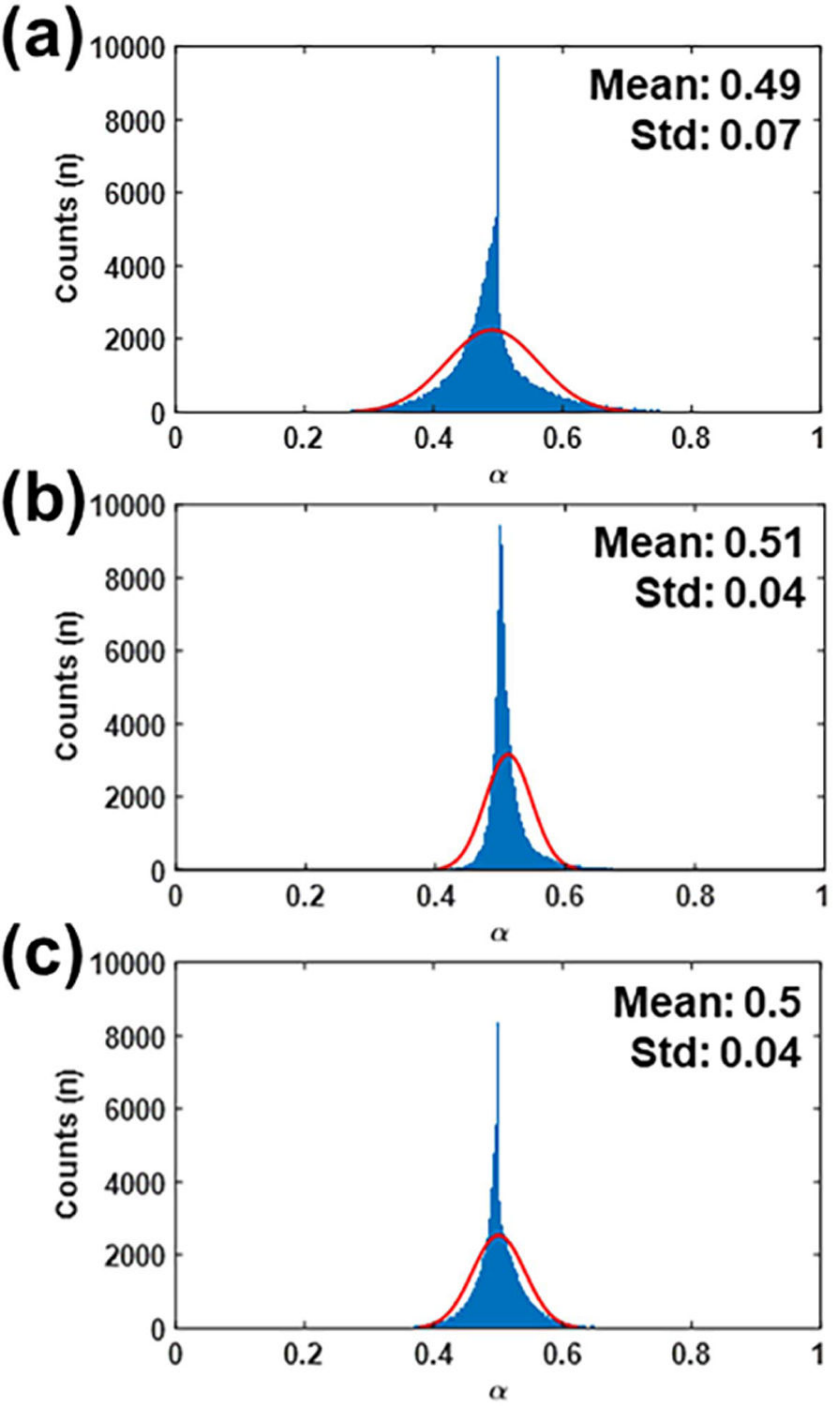
Histogram with a normal distribution fit of *α* acquired from (a) simulation, (b) CAM, and (b) mouse studies. The red lines represent fitted line.

Fig. 12 shows the histogram with a normal distribution fit of *α* values acquired under simulation, CAM, and mouse experiments. The mean and standard deviation of *α* were 0.49 ± 0.07, 0.51 ± 0.04, and 0.5 ± 0.04 for simulation, CAM, and mouse, respectively. Therefore, the value of *α* was fairly consistent in our study.

The filtering-based bubble separation is an additional postprocessing step to enhance the localization performance for situations with high-concentration MBs. MUTE is not postprocessing-based and directly uncouples MBs in a high MB density environment using null transmission. We used three filter banks to balance the tradeoff between MB separation performance and added computational costs associated with MB separation. As shown in Fig. 6(e) and (f), we elected to combine MB separation with MUTE because we found that the gain in MB localization performance was substantial with MB separation. For example, the maximum NCC coefficient was improved from 0.51 to 0.55 from PW to MUTE without bubble separation, and from 0.51 to 0.59 from PW to PW with bubble separation. Nevertheless, it is also clear that when combining MB separation with MUTE, ULM performance was the best.

From the *in vivo* CAM and mouse brain imaging results, we did not find MUTE-ULM to be particularly more susceptible to tissue motion than conventional PW-ULM. In this study, MUTE-ULM used five transmissions at each steering angle with a pulse repetition frequency of 22.7 kHz and achieved an effective frame rate of 500 Hz, which is similar to most ULM applications with compounding PW imaging as the detection sequence. However, it is unclear whether tissue motion would compromise the MB uncoupling performance (e.g., the null subtraction steps) for MUTE. We speculate that MB movement within the bloodstream (especially for large vessels with high flow speed) may be a more significant source of motion than tissue, but again it is unclear to what extent would it affect the MUTE performance within the context of MB uncoupling. For compounding PW, it is understood that such motion may decrease the coherence of MB signals from each transmission angle, which leads to suboptimal transmit focusing synthetization that deteriorates the MB signal (e.g., blurring). We expect a similar issue with MUTE but again based on the *in vivo* study results, we do not think MUTE is more susceptible to tissue or MB motion than conventional PW imaging.

For practical implementations of MUTE, there are several tradeoffs that need to be balanced. First, the number of nulls that can be simultaneously generated has a positive relationship with the imaging frame rate: the more the number of nulls that can be simultaneously generated in the FOV to cover a large region, the less the number of transmissions needed for constructing the full FOV, and hence the higher the imaging frame rate. In this article, we used eight elements to generate each null (31 nulls total for the 128-element transducer). The resulting image frame rate was 500 Hz (after three angle compounding and four lateral translations to cover the full FOV), which is adequate for most ULM applications. The second tradeoff is between the width and the number of the acoustic nulls and their relationship to the imaging frame rate. In theory, a larger number of elements could generate narrower nulls with lower acoustic pressure. However, the more the elements used for each null, the less the number of nulls that can be generated simultaneously, and consequently the slower the frame rate. In this study, the width of the acoustic nulls was measured at approximately 0.65*λ* (50 *μ*m). Therefore, MBs with spacing less than 0.65*λ* (50 *μ*m) may not get uncoupled effectively. Finally, one needs to note that the effect of uncoupling by MUTE is more significant in the lateral direction than in the axial direction. Although steering may introduce uncoupling in the axial direction, due to the limited steering angle that is achievable on conventional ultrasound transducers, MUTE still largely promotes MB separation along the lateral direction. Nevertheless, because the lateral resolution is intrinsically inferior to axial resolution in ultrasound imaging, improvement in the lateral direction is necessary and significant for robust ULM.

In contrast to a recent study using activatable phase-changing nanodroplets for super-resolution imaging (see [27]), MUTE “silences” MBs to enhance the imaging performance, while the nanodroplets-based method “evokes” MBs. Unlike optical contrast such as fluorophores, MBs cannot be switched between ON and OFF status repeatedly. For example, once a nanodroplet gets activated to become an MB (i.e., switch ON), one can only switch them OFF by disruption (e.g., using high MI acoustic pulses), after which it is impossible to switch them back ON again. However, one advantage of using phase-changing nanodroplets over conventional MBs is that one can selectively activate nanodroplets in a controlled-release fashion where local MB concentration can be maintained at a certain level that facilitates robust ULM performance (e.g., low MB density that facilitates better MB localization in larger vessels). This is similar to MUTE where local MB concentration is reduced by the acoustic nulls that suppress the MB signal. One downside of nanodroplets is that they can extravasate to extravascular space due to their small size, which confounds the microvascular imaging results. In addition, nanodroplets are not approved by the FDA and are not immediately available to be used for clinical applications. Nevertheless, nanodroplets do offer an enticing option for imaging beyond the microvasculature and providing new functional biomarkers such as vessel leakiness and targeted molecular imaging.

## V. CONCLUSION

In this article, we demonstrate a new imaging sequence (MUTE—MB uncoupling via transmit excitation) that uses the principles of acoustic null and image subtraction to uncouple and separate spatially overlapping MB signals that are otherwise unlocalizable in conventional ULM imaging. We showed that MUTE could significantly improve the MB localization efficacy and imaging speed of ULM under high MB concentrations. These results indicate that MUTE has the potential to alleviate the issues of long data acquisition time and low temporal resolution of ULM, which is significant for practical implementations of ULM, especially under clinical settings.
